# Supplementary material for: Latent classes of mental health disorders and their associations with polymorphisms of 5HTTLPR and BDNF in a Chilean primary care population
Source: Glob Ment Health (Camb). 2025 Oct 10;12:e144. doi: 10.1017/gmh.2025.10062 (PMC12720213; doi:10.1017/gmh.2025.10062)
Supplement: Moraga-Escobar et al. supplementary material [file S2054425125100629sup001.docx]

**Appendix 1. Prevalence of lifetime mental health diagnoses in the total sample and in the latent classes**

|  |  | **Total**  **(N=789)** |  | **Latent Class 1**  **(Higher psychiatric comorbidity)**  **(n=161)** | **Latent Class 2**  **(Lower psychiatric comorbidity)**  **(n=628)** | **p** |
| --- | --- | --- | --- | --- | --- | --- |
| **Agoraphobia** | No | 735 (93.2) |  | 111 (68.9) | 624 (99.4) | **<0.001** |
|  | Yes | 54 (6.8) |  | 50 (31.1) | 4 (0.6) |  |
| **Generalised anxiety** | No | 697 (88.3) |  | 109 (67.7) | 588 (93.6) | **<0.001** |
|  | Yes | 92 (11.7) |  | 52 (32.3) | 40 (6.4) |  |
| **Bipolar depression** | No | 773 (98.0) |  | 145 (90.1) | 628 (100.0) | **<0.001** |
|  | Yes | 16 (2.0) |  | 16 (9.9) |  |  |
| **Dysthymia** | No | 723 (91.6) |  | 113 (70.2) | 610 (97.1) | **<0.001** |
|  | Yes | 66 (8.4) |  | 48 (29.8) | 18 (2.9) |  |
| **Recurrent depression** | No | 727 (92.1) |  | 125 (77.6) | 602 (95.9) | **<0.001** |
|  | Yes | 62 (7.9) |  | 36 (22.4) | 26 (4.1) |  |
| **Simple depression** | No | 648 (82.1) |  | 119 (73.9) | 529 (84.2) | **0.004** |
|  | Yes | 141 (17.9) |  | 42 (26.1) | 99 (15.8) |  |
| **Dissociative disorders** | No | 717 (90.9) |  | 112 (69.6) | 605 (96.3) | **<0.001** |
|  | Yes | 72 (9.1) |  | 49 (30.4) | 23 (3.7) |  |
| **Social phobia** | No | 703 (89.1) |  | 105 (65.2) | 598 (95.2) | **<0.001** |
|  | Yes | 86 (10.9) |  | 56 (34.8) | 30 (4.8) |  |
| **Specific phobias** | No | 513 (65.0) |  | 30 (18.6) | 483 (76.9) | **<0.001** |
|  | Yes | 276 (35.0) |  | 131 (81.4) | 145 (23.1) |  |
| **Mania** | No | 767 (97.2) |  | 139 (86.3) | 628 (100.0) | **<0.001** |
|  | Yes | 22 (2.8) |  | 22 (13.7) |  |  |
| **Panic disorder** | No | 786 (99.6) |  | 161 (100.0) | 625 (99.5) | 1.000 |
|  | Yes | 3 (0.4) |  |  | 3 (0.5) |  |
| **Post-traumatic stress** | No | 674 (85.4) |  | 90 (55.9) | 584 (93.0) | **<0.001** |
|  | Yes | 115 (14.6) |  | 71 (44.1) | 44 (7.0) |  |
| **Somatomorphic disorders** | No | 673 (85.3) |  | 105 (65.2) | 568 (90.4) | **<0.001** |
|  | Yes | 116 (14.7) |  | 56 (34.8) | 60 (9.6) |  |
| **Obsessive compulsive disorder** | No | 788 (99.9) |  | 160 (99.4) | 628 (100.0) | 0.204 |
|  | Yes | 1 (0.1) |  | 1 (0.6) |  |  |
| **Eating disorder** | No | 785 (99.5) |  | 158 (98.1) | 627 (99.8) | **0.028** |
|  | Yes | 4 (0.5) |  | 3 (1.9) | 1 (0.2) |  |
| **Schizophrenia** | No | 779 (98.7) |  | 155 (96.3) | 624 (99.4) | **0.007** |
|  | Yes | 10 (1.3) |  | 6 (3.7) | 4 (0.6) |  |
| **Psychosis** | No | 783 (99.2) |  | 161 (100.0) | 622 (99.0) | 0.608 |
|  | Yes | 6 (0.8) |  |  | 6 (1.0) |  |
| **Schizoaffective disorder** | No | 788 (99.9) |  | 160 (99.4) | 628 (100.0) | 0.204 |
|  | Yes | 1 (0.1) |  | 1 (0.6) |  |  |

**
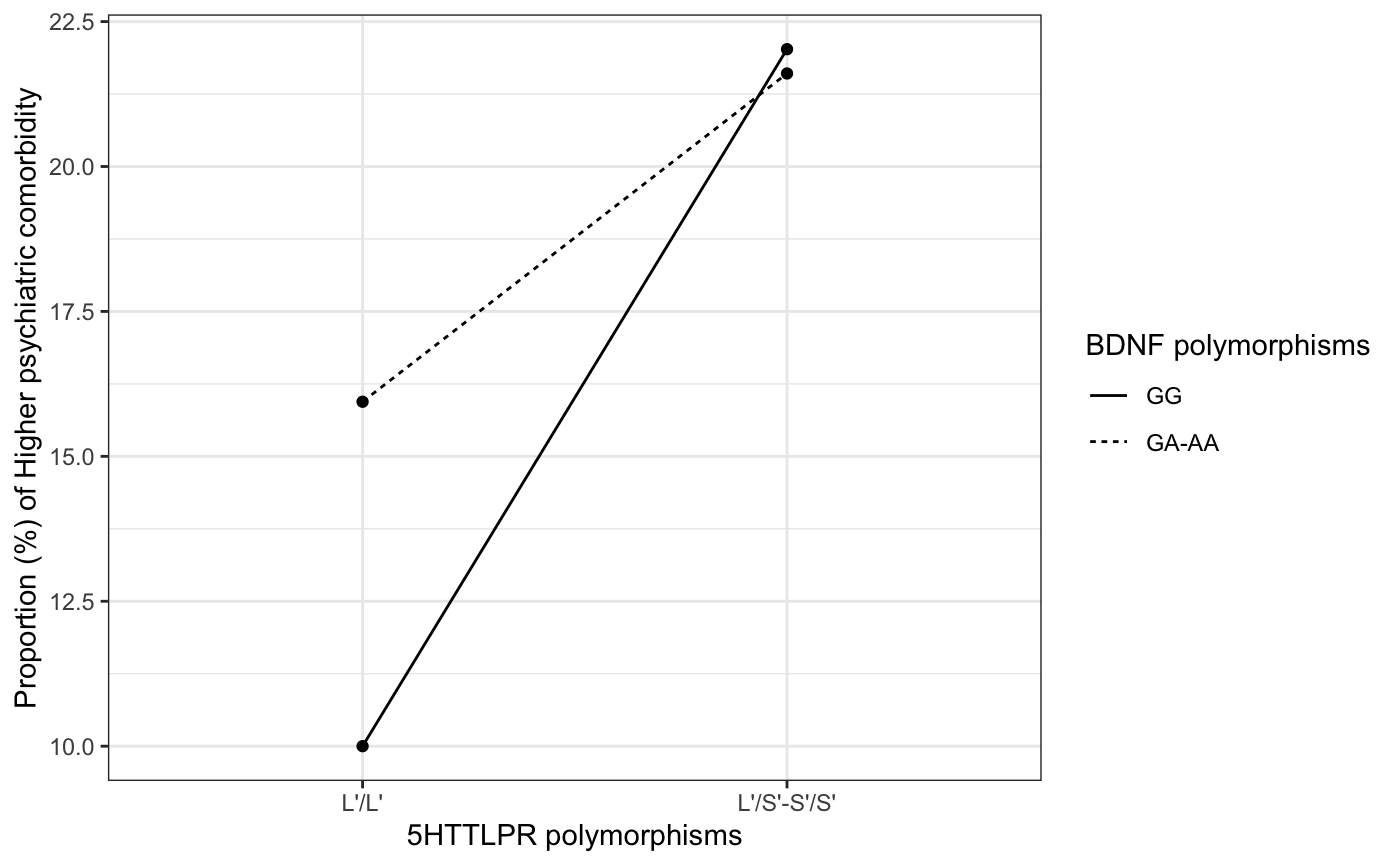
**

**Appendix 2.** Changes in the proportion of patients in the highest psychiatric comorbidity group based on BDNF x 5HTTLPR genetic polymorphism
